# Catheter‐related blood stream infection caused by *Mycobacterium chelonae* in a child with myeloid leukemia associated with Down syndrome

**DOI:** 10.1002/ccr3.3646

**Published:** 2020-12-18

**Authors:** Tomoko Fujikawa, Suguru Uemura, Yuya Aoto, Yoshinori Nambu, China Nagano, Naoko Nakatani, Nanako Nino, Nobuyuki Yamamoto, Takeshi Mori, Noriyuki Nishimura, Kazumoto Iijima

**Affiliations:** ^1^ Department of Pediatrics Kobe University Graduate School of Medicine Kobe Japan; ^2^ Department of Hematology and Oncology Kobe Children’s Hospital Kobe Japan; ^3^ Kobe University Graduate School of Health Sciences Kobe Japan

**Keywords:** catheter‐related blood stream infection, central venous catheter, mycobacterium chelonae, rapidly growing nontuberculous mycobacteria

## Abstract

Rapidly growing nontuberculous mycobacteria should be considered if GPRs gram‐positive rods are detected in blood cultures 2‐3 days after the blood sample collection.

## Graphical abstract

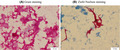

## 1 INTRODUCTION

Rapidly growing nontuberculous mycobacteria (RGM) are rare causative pathogens of catheter‐related blood stream infection (CRBSIs) and can sometimes be misdiagnosed as gram‐positive rods (GPRs). Therefore, RGM should be considered if GPRs are detected in blood cultures 2‐3 days after sample collection.

A central venous catheter (CVC), which is necessary for the treatment of children with cancer,^1^ often leads to complications such as infections, occlusion, breakage, and thrombosis, not only at the time of insertion but also during use.^1^, ^2^ The catheter‐related blood stream infection (CRBSI) is a common complication in pediatric cancer patients. It has been estimated that 14%–51% of CVCs implanted in children with cancer may be complicated by bacteremia.^1^ The most common organisms that cause CRBSIs are coagulase‐negative staphylococci, *Staphylococcus aureus*, *Enterococcus* spp., *Escherichia coli*, *Klebsiella* spp., and *Candida* spp.^1^, ^2^

Rapidly growing nontuberculous mycobacteria (RGM) are defined as nontuberculous mycobacteria (NTM) that persist in a variety of environmental sources, such as water, soil, and aquatic animals, and that form mature colonies on solid agar within 7 days.^3^ More than 70 RGM species have been reported so far.^3^ NTM can cause lymphadenopathy, skin and soft tissue infections, pulmonary disease, and disseminated disease.^3^ In hospitals, RGM may also be responsible for outbreaks of infections that originate from contaminated medical equipment and water.^3^ However, RGM are considered a rare cause of CRBSIs.^4^, ^5^, ^6^ Herein, we report a patient of a myeloid leukemia associated with Down syndrome (ML‐DS) who developed a CRBSI caused by *Mycobacterium chelonae*.

## 2 CLINICAL CASE PRESENTATION

A 43‐month‐old female patient with trisomy 21 was admitted to our hospital due to fever. She was diagnosed with ML‐DS at 24 months old. She achieved complete remission (CR) after the first course of induction chemotherapy and remained in CR during treatment with additional chemotherapy. However, she relapsed 1 month after the completion of chemotherapy. She received reinduction chemotherapy and achieved a second CR after the first course of reinduction chemotherapy. She underwent bone marrow transplantation (BMT) in the second CR. However, the second relapse occurred after BMT at 37 months old. The following chemotherapy which consisted of azacitidine reduced leukemic cell counts in the bone marrow, and she achieved a third CR.^7^ She was closely being followed up at the outpatient department without further intervention. At the time of admission, she was on sulfamethoxazole/trimethoprim and voriconazole for infection prophylaxis and was also prescribed prednisolone (0.25 mg/kg/day) for the treatment of chronic graft‐versus‐host disease (GVHD). Since her peripheral intravenous catheterization was very difficult to access, a Hickman‐type CVC with a double lumen accessed from the right internal jugular vein through a subcutaneous tunnel was used. This CVC was inserted at the time of first relapse at 31 months old.

At admission, her general appearance was not good, she had a high temperature (39.7℃), her blood pressure was 98/60 mm Hg, pulse 162 /min, respiratory rate of 36 /min with an O2 saturation of 100% on room air. She vomited twice in the consultation room. No remarkable physical findings except slightly increased bowel sounds and rash caused by chronic skin GVHD that extended from the trunk to the legs were observed. No tenderness, redness, or swelling was observed in the region where the CVC was inserted or in the subcutaneous tunnel. Laboratory examinations revealed a white blood cell count of 3,800 /µL with 66.0% neutrophil and CD4‐positive cell count of 317 /µL, and a hemoglobin level of 10.9 g/dL. Although the platelet count was 23,000 /µL at admission, her platelet counts changed from 20,000 to 50,000 /µL during the outpatient follow‐up period. CRP was 5.69 mg/dL. Immunoglobulin G (IgG), IgA, and IgM were 729, 21, and 50 mg/dL, respectively.

**Figure 1:**
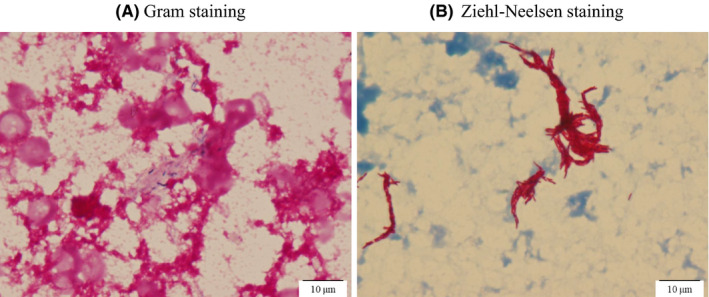
*Mycobacterium chelonae* (A) Gram staining; gram‐positive rod was detected (B) Ziehl‐Neelsen staining

Initially, bacterial colitis was suspected as the cause of fever, and cefmetazole at a dose of 100 mg/kg/d was initiated through the CVC, which resolved her high‐grade fever. On day 3 of hospitalization, gram‐positive rods (GPRs) were detected in the blood culture collected from the CVC at the time of admission. We had failed to collect two sets of blood culture samples at admission. Since she remained afebrile and the contamination was suspected, the antibiotics treatment regimen did not be changed, another two sets of blood culture samples were collected from the CVC and peripheral blood, and the CVC was left in place. However, on day 5 of hospitalization, GPRs were again detected from another two sets of blood culture samples, and *M. chelonae* was isolated from the blood culture sample collected at admission (Figure 1).

Blood was collected in BD BACTEC^TM^ Peds Plus^TM^/F Culture Vials (Plastic) (Becton Dickinson, Sparks, MD, USA). Blood cultures were incubated in the BD BACTEC FX system. *M. chelonae* was identified by matrix‐assisted laser desorption ionization‐time of flight mass spectrometry using a MALDI Biotyper (Bruker Daltonik, Germany).

An intravenous line was established, and the antibiotics regimen was switched from cefmetazole to imipenem/cilastatin (100 mg/kg/day), clarithromycin (15 mg/kg/day), and amikacin (8 mg/kg/day), and the CVC was removed on the following day. After the susceptibility test confirmed the presence of *M. chelonae*, amikacin was stopped and tobramycin was initiated at a dose of 5 mg/kg/day (Table 1). The combination of antibiotics including imipenem/cilastatin, clarithromycin, and tobramycin was continued until day 27 of hospitalization. After the completion of antibiotic treatment, no recurrence occurred. No evidence of abscess formation was observed on imaging during her admission including brain MRI, chest and abdominal CT, and cardiac ultrasonography. There was no evidence of *M. chelonae* in the blood culture samples collected on day 15 of hospitalization. The growth of GPRs was detected in the blood culture sample from the CVC more than 2 hours earlier than that obtained from the peripheral blood on day 3 of hospitalization. Therefore, she was diagnosed as a CRBSI caused by *M. chelonae*.

**Table 1:** The drug susceptibility profile of *Mycobacterium chelonae*

Antibiotics	MIC	Susceptibility
Amikacin	<8	S
Tobramycin	<1	S
Clarithromycin	<1	S
Ciprofloxacin	1	S
Sulfamethoxazole‐ Trimethoprim	>4	R
Imipenem	8	I
Meropenem	>16	R
Linezolid	16	I
Azithromycin	2	S
Doxycycline	<1	S
Moxifloxacin	2	I

Abbreviations: I: intermediate; MIC, minimum inhibitory concentration; R, resistant; S, susceptible.

## 3 DISCUSSION

We herein reported a patient with ML‐DS who developed a CRBSI due to *M. chelonae*. *M. fortuitum*, *M. abscessus*, and *M. chelonae* are well‐known RGM, which appear similar to GPRs by Gram staining and can grow as small colonies on sheep blood and chocolate agar. However, these characteristics depend on the Gram staining technique, blood culture media, and culture conditions such as temperature.^5^ Therefore, the early diagnosis of CRBSIs caused by RGM is challenging. In a report of six patients, Hawkins C, et al indicated that CRBSIs caused by RGM in all cases were initially considered as the growth of gram‐positive bacilli by Gram staining of the aerobic blood culture broths after 2‐5 days of incubation.^5^ RGMs have also been reported to be misidentified as *Corynebacterium* spp, *Rhodococcus* spp, and *Nocardia* spp.^8^, ^9^

We summarized the current literature of CRBSIs caused by RGM in pediatric hematology and oncology patients. To do this, we used the PubMed database to search for English language articles related to CRBSIs cause by RGM in children.^4^, ^6^, ^8^, ^36^ (Table 2). Most cases were caused by *M fortuitum*, *M. chelonae*, or *M*. *mucogencum* among RGM. Two reports described the outbreak of bloodstream infection by RGM among different pediatric hematology and oncology departments.^17^, ^22^ Both of those were caused by *M. mucogenicum,* and one report was related to the contaminated water supply in the institute.^17^ Two patients with trisomy 21 were diagnosed as CRBSI caused by RGM.^16^, ^31^ Fourteen patients underwent allogeneic hematopoietic stem cell transplantation before the occurrence of CRBSIs caused by RGM. Eight patients experienced the recurrence of RGM infections. Two patients experienced second RGM infections caused by another *Mycobacterium* species. One patient with acute myeloid leukemia experienced the bacteremia caused by *M mucogenicum*, and in this case, multiple lung nodules on chest CT were observed. The removal of CVC and 4 total weeks of antibiotic treatment led to resolution. However, 2 months after the completion of antibiotic therapy, the patient experienced another episode of bacteremia caused by *M aurum/neoaurum*.^26^ Another patient with acute lymphoblastic leukemia experienced an infection at the CVC exit site caused by *M. fortuitum,* and 7 months later, a totally implanted device pocket infection caused by *M. abscessus* occurred.^6^ No patients died of CRBSIs caused by RGM, although some patients died due to the progression of their malignancies.

**Table 2:** The clinical characteristics of 93 children with catheter‐related blood stream infection caused by rapid growing mycobacterium

Characteristics	Number
Median age years (range)	4 (0‐18)
Sex	
Male	56
Female	30
N/A	7
Underlying disease	
Trisomy 21	2
Klinfelter syndrome	1
Type of CRBSI	
Disseminated	19
CLABSI	49
Exit site	17
Tunnel tract infection	6
TID pocket	2
Hematology/Oncology diagnosis	
Hematological malignancies	56
Solid tumors	31
Others^a^	6
Type of Catheter	
Hickman	49
Brobiac	7
Port	8
N/A	29
HSCT	14
Organism	
*Mycobacterium chelonae*	26
*Mycobacterium fortuitum*	25
*Mycobacterium mucogenicum*	22
*Mycobacterium abscessus*	5
*Mycobacterium neoaurum*	4
*Mycobacterium fortuitum/chelonae complex*	4
*M. chelonae‐abscessus*	2
*M. immunogenum*	2
*M. aurum*	1
*M. hackensackense*	1
*M. flurantbenivorans*	1
*M. lacticola*	1
*M. smegmatis*	1
*M. aurumu/neoaurum*	1
Removal of CVC	
Yes	81
No	5
N/A	8
Use of antibiotics	
Yes	70
No	10
N/A	13
Recurrence	8

Abbreviations: CLABSI, central line‐associated bloodstream infection; CRBSI, catheter‐related blood stream infection; CVC, central venous catheter; HSCT, hematopoietic stem cell transplantation; TID, totally implantable device.

^a^ Two patients: aplastic anemia, 2 patients: Wiskott‐Aldrich syndrome, 1 patient: hemophilia, 1 patient: thalassemia.

Apiwattankul N, et al reported that the incidence of RGM infections in children with cancer was 0.4 cases/100,000 patient days.^6^ A retrospective analysis at a single center in Taiwan revealed that there was one patient with an CRBSI caused by RGM among 259 cases of CRBSIs in pediatric cancer patients.^4^ Because of the low incidence of RGM infections in pediatric cancer patients, we did not consider the possibility of RGM as the etiology of CRBSIs. Consequently, in the present case, the change of the antibiotics and the removal of CVC were delayed even though the GPRs were detected in a blood culture collected on day 3 of hospitalization.

In a study of adult cancer patients treated at a large center, almost half of the patients with RGM infections had pulmonary disease.^37^ In contrast, pulmonary infections are less common in children,^6^ which might be associated with the higher rate of pulmonary comorbidities such as chronic obstructive pulmonary disease in adults compared with children. A report from St Jude Children's Research Hospital revealed that approximately half of the patients (42 patients) developed CRBSIs among 85 episodes with cancer diagnosed with RGM infections.^6^ Furthermore, exit‐site infections, tunnel tract infections, and infections from other implantable devices were found in 17, 6, and 2 patients, respectively.^6^ These results might reflect the importance of CVC for the treatment of children with cancer compared with adults. Similar to our case, most patients in that study had a neutrophil count of more than 500/µL at the onset of RGM infection. In our case, her CVC had been in place for approximately 12 months. Therefore, in our case, the long‐term CVC placement might have been associated with the development of CRBSI.

Although no controlled clinical trials have investigated the treatment approaches for CRBSIs caused by RGM, combination antimycobacterial therapy is recommended since macrolide monotherapy may lead to the emergence of resistance for antibiotics.^38^, ^39^ The removal of CVC is also a very important intervention for the treatment of CRBSIs caused by RGM. The removal of CVC and appropriate antibiotics is required to achieve a successful outcome.^5^ Treatment failure can occur if the infected CVC is retained even when the appropriate antibiotics are administered.^5^ According to our review, most patients underwent the removal of CVC, while CVC was not removed in five cases. Two patients without the removal of CVC experienced the recurrence of CRBSI caused by RGM.^7^, ^36^ The removal of CVC followed by the antibiotic treatment resolved the second CRBSI caused by RGM. Therefore, the removal of CVC is likely necessary for the resolution of CRBSIs caused by RGM.

In summary, RGM are rare causative pathogens of CRBSIs and can sometimes be mistaken for GPRs. Therefore, RGM should be considered if GPRs are detected in blood cultures 2‐3 days after sample collection. Furthermore, the removal of CVC is a crucial intervention for the treatment of CRBSIs caused by RGM.

## CONFLICT OF INTEREST

None declared.

## AUTHOR CONTRIBUTIONS

TF, SU, YA, YN, CN, NN, NN, NY, and TM: participated in patient's evaluation and treatment. TF, SU, NN, and KI: reviewed and revised the manuscript. All authors read and approved the final manuscript.

## ETHICS STATEMENT

Written informed consent for publication of the case and images was obtained from the patient prior to the writing of this case report.

## Data Availability

The data that support the findings of this study are available on request from the corresponding author. The data are not publicly available due to privacy or ethical restrictions.
